# A cross-sectional study assessing determinants of the attitude to the introduction of eHealth services among patients suffering from chronic conditions

**DOI:** 10.1186/s12911-015-0157-3

**Published:** 2015-04-19

**Authors:** Mariusz Duplaga

**Affiliations:** Department of Health Promotion, Institute of Public Health, Faculty of Health Sciences, Jagiellonian University Medical College, Grzegorzecka Str. 20, 31-531 Krakow, Poland

**Keywords:** eHealth, Health care, Chronic disease, Internet use, Computer literacy

## Abstract

**Background:**

Provision of care to patients with chronic diseases remains a great challenge for modern health care systems. eHealth is indicated as one of the strategies which could improve care delivery to this group of patients. The main objective of this study was to assess determinants of the acceptance of the Internet use for provision of chosen health care services remaining in the scope of current nationwide eHealth initiative in Poland.

**Methods:**

The survey was carried out among patients with diagnosed chronic conditions who were treated in three health care facilities in Krakow, Poland. Survey data was used to develop univariate and multivariate logistic regression models for six outcome variables originating from the items assessing the acceptance of specific types of eHealth applications. The variables used as predictors were related to the sociodemographic characteristics of respondents, burden related to chronic disease, and the use of the Internet and its perceived usefulness in making personal health-related decisions.

**Results:**

Among 395 respondents, there were 60.3% of Internet users. Univariate logistic regression models developed for six types of eHealth solutions demonstrated their higher acceptance among younger respondents, living in urban areas, who have attained a higher level of education, used the Internet on their own, and were more confident about its usefulness in making health-related decisions. Furthermore, the duration of chronic disease and hospitalization due to chronic disease predicted the acceptance of some of eHealth applications. However, when combined in multivariate models, only the belief in the usefulness of the Internet (five of six models), level of education (four of six models), and previous hospitalization due to chronic disease (three of six models) maintained the effect on the independent variables.

**Conclusions:**

The perception of the usefulness of the Internet in making health-related decision is a key determinant of the acceptance of provision of health care services online among patients with chronic diseases. Among sociodemographic factors, only the level of education demonstrates a consistent impact on the level of acceptance. Interestingly, a greater burden of chronic disease related to previous hospitalizations leads to lower acceptance of eHealth solutions.

**Electronic supplementary material:**

The online version of this article (doi:10.1186/s12911-015-0157-3) contains supplementary material, which is available to authorized users.

## Background

In recent years, the provision of care to patients with chronic conditions has become one of the greatest challenges faced by health care systems. Patients with chronic diseases require care for prolonged periods. Estimates indicate that in modern societies up to 40% of people suffer from chronic conditions [1]. Furthermore, expenditures related to care provided to patients with chronic diseases are a significant part of health care budgets [2].

Chronic care requires regular interactions between patients and health care systems. Patient involvement in the monitoring and treatment of disease is of crucial importance for the effectiveness of the therapy [3,4]. The growing incidence of chronic diseases can lead to a shortage of health care resources available to patients and their families. All these circumstances trigger intensive searches for new models of care which would enable an adequate and cost-effective support offered to patients with chronic diseases. Development of eHealth environment is frequently indicated as a promising response to challenges associated with chronic care [5-9].

The performance of the health care system in Poland is a continuous source of frustration for patients. A survey conducted among Polish citizens in 2012 revealed that 78% respondents were not satisfied with the health care system. The main complaints were related to patients having a limited access to diagnostic tests and specialty care [10]. A regular nationwide survey “Social Diagnosis” carried out in 2013 also showed that according to 28% of households, the fulfilment of health care needs worsened in last year, and only 2% of respondents declared an improvement of the situation [11]. Reforms of the health care system have lagged behind the political and economic transformation of the early 1990s. More profound changes to the health care system were initiated in the late 1990s. Unfortunately, consecutive governments have not been consistent in maintaining the direction of the reforms. Initially, the responsibility for organizing the provision of health care services was delegated to a network of regional health funds. The next government reversed the regional system of health care funding and, in 2003, introduced a centralized system based on the National Health Fund [12]. Although funding of the health service has increased considerably in the last decade, it remains low in comparison to other EU member states. According to WHO, Polish health expenditure per capita measured in US dollars Purchasing Power Parity (PPP) in 2008 was among the lowest in Europe [13]. Growing expectations of patients and society related to health care services overlap with a general shortage of available resources in the health care system [14,15]. Recent attempts to optimize the health service reimbursement system, the introduction of diagnosis-related group (DRG)-based reimbursement and new rules for setting lists of reimbursed pharmaceuticals have not translated into a better financial standing for health care institutions and patient satisfaction [16]. To sum up, the health care system suffers from many problems, and remedial actions to date have not been fully effective [17]. This has led to a continued search for new strategies which would result in the optimization of care delivery. The use of information and communication technologies (ICT) in health care is perceived as an approach which has a potential to increase efficiency and cost-effectiveness of services.

Indeed, for at least the last two decades there has been a growing trend to increase the implementation of ICT systems in an attempt to support both health care providers and patients. These efforts have been intensified after Poland’s accession to the European Union; a part of the structural funds available to Poland have been directed to the public domain for investing in ICT infrastructure. There are also ambitious plans to develop an advanced information infrastructure in health care [18,19]. Expanding the use of eHealth systems in order to support patients and citizens has become one of key elements of the strategy adopted by the Polish government [20]. The applications enlisted on the priority list include Internet-based medical record, e-prescribing, online access to results of laboratory tests, and making appointments to see physicians [20].

Nowadays, it is obvious that eHealth development is a participatory process and depends strongly on the involvement of all relevant stakeholders [21]. The rationale for developing specific types of eHealth solutions cannot rely on administrative decisions only, but must also address the needs and attitudes of recipients of care services. Thus, plans for the implementation of eHealth applications in chronic care should consider the acceptance and preparedness of patients. Assessing patients’ opinions could provide important information about the acceptance of specific solutions. Additionally, potential factors influencing these opinions should be studied and considered in the process of making decisions about the priorities for eHealth implementation when formulating national level policies.

The acceptance of technology is usually understood as both the intention to use it and its actual use. Strategies of the assessment of eHealth acceptance stem from theories related to behavioural change and technology acceptance. Among them, the technology acceptance model (TAM) [22] and the unified theory of acceptance and use of technology (UTAUT) [23] were most frequently used in relation to eHealth acceptance among health professionals and patients. The TAM model emphasizes two aspects: perceived usefulness and perceived ease of use [24]. In turn, UTAUT is based on four determinants of intention and usage: performance expectancy, effort expectancy, social influence and facilitating conditions. This theory also anticipates behavioural intention and observable use behaviour may be influenced by four moderators including gender, age, experience and voluntariness of use [23].

It also seems that eHealth acceptance among patients has not been addressed so frequently by researchers as its acceptance among professionals. Most reports on eHealth acceptance by physicians published in the last decade applied the two theoretical models mentioned above [25-27].

In their study of patient’s acceptance of provider-delivered eHealth, Wilson et al. used the TAM model and the motivational model [28]. The TAM model was also used in the assessment of the acceptance of an application for Internet-based patient-physician communication [29] and an application designed for patients with HIV/AIDS [30]. The UTAUT theory was used by Beenkens to assess eHealth acceptance among patients with venous thrombosis [31]. This author demonstrated that patient performance and effort expectancies have a strong influence on behavioural intentions to use eHealth technology; additionally a quality of health care measure should be included in the assessment.

Ehealth acceptance is a complex concept related to individual preferences and attitudes. It may be of key importance for the successful implementation of eHealth solutions. Apart from eHealth acceptance, the concept of “eHealth readiness” related to the ability of potential users or health care organizations to benefit from eHealth applications is used by researchers [32-34]. Insights into eHealth readiness may facilitate proceeding efficiently with the development of an eHealth environment.

The main objective of this study was to assess opinions of patients with chronic conditions regarding the feasibility of eHealth to support health care activities in the context of national initiatives focused on the formation of an eHealth environment. The paper presents results of the assessment of patient perception of concrete types of services provided via the Internet. An assessment was also carried out with the intention of yielding guidance for representatives of governmental bodies involved in decision-making in relation to the development of an eHealth domain on the national level. Advanced eHealth tools are not commonly available for end-users in Poland, and patients with chronic diseases may still be perceived as an eHealth-naive population. Thus, the analysis is based on items asking for opinions about the possibility of using the Internet for the provision of health care services rather than their actual use or perceived usefulness originating from real experience. The results of the survey conducted among patients with chronic diseases receiving care from three medical centres in the urban area of Krakow, Poland, form the basis for this analysis.

It was not the aim of this paper to provide a comprehensive view of eHealth acceptance among patients with chronic conditions. Instead, the paper focuses on specific eHealth applications enabling the provision of health services essential in chronic care, in relation with policies developed on the national level. Thus, the term ”acceptance” is used throughout the paper only in relation to concrete types of eHealth solutions. It is applied to designate the respondents’ positive opinion about the possibility of providing of specific health care service online.

## Methods

### Overview

A cross-sectional survey was performed in a convenience sample of 524 patients with chronic diseases who remained under the care of three medical centres located in Krakow, Poland. The study was conducted from December 2011 to April 2012 until 400 completed questionnaires were returned. The survey focused on the assessment of the opinions of patients suffering from long-term medical conditions about the feasibility of eHealth-based services for care provision. In this paper, the results of the analysis of factors influencing the acceptance of specific eHealth applications relevant to chronic care are presented. The selection of these applications was dictated by their relationship with national policies of eHealth development and current activities in this field in Poland.

### Survey questionnaire

The questionnaire developed for the study covered aspects related to the burden of disease, the use of information technologies (IT), health-related use of the Internet, eHealth literacy (eHEALS scale) [35], the acceptance of the use of the Internet and related technologies for the provision of health-related services, and sociodemographic characteristics of the respondents. The key areas covered by the questionnaire stemmed from the review of international references related to the assessment of the eHealth acceptance and use by target audiences. The questionnaire was piloted in a group of 10 patients. As a result, some items were removed as they were deemed to be redundant, or modified to provide a better understanding.

The final version of the questionnaire included 73 items. Questions asking for the respondents’ opinions used the five-point Likert scale (from strongly disagree to strongly agree with neutral in the middle position). The questionnaire also included items assessing the frequency of specific events or activities (relevant frequency scales were assigned) and the respondents’ use of computers and the Internet, also in relation to general and health-related activities with dichotomous (yes/no) response options. For questionnaire items used for the analysis presented in the paper see Additional file 1.

### Study setting and participants

The questionnaires were distributed to patients admitted to hospital or attending an ambulatory visit in three healthcare facilities in the Krakow area: the Department of Metabolic Diseases and the Department of Pulmonology, both part of the Jagiellonian University Medical College, and the Jozef Dietl Municipal Hospital in Krakow. The survey was carried out among patients suffering from medical conditions requiring long-term support from the health care system. Thus, only patients with an established diagnosis of a chronic disease with an established treatment were recruited to the survey. Patients who were hospitalized or admitted to polyclinics for diagnosis of new symptoms were not included in the study, unless they had previously been diagnosed with a chronic disease.

### Study protocol

The respondents were first informed about the objectives and scope of the survey, and asked for their consent to be included in the study and for processing of the data included in the questionnaire. Questionnaires were distributed to the respondents by interviewers employed and trained for the study, recruited from the medical personnel of the health care centres participating in the study. The study protocol was approved by the Bioethical Committee at the Jagiellonian University (decision No. KBET/107/B/2011 dated 30 June 2011).

### Measures

#### Sociodemographic characteristics

The respondents were asked to provide information about their gender, age, education level, and place of residence. The item asking about the respondents’ education included 9 options from basic to university level, specific to the Polish education system. Nine levels of education included in the questionnaire were collapsed into three categories: (1) education level lower than upper secondary according to the International Standard Classification of Education (ISCED) [36], (2) education level including upper secondary to post-secondary non-tertiary according to ISCED, and (3) education level covering all levels according to ISCED higher than post-secondary non-tertiary. The place of residence was initially assigned five options: rural, urban with <10 000, urban 10 000 – 100 000, urban 100 000 – 500 000, and urban with >500 000 inhabitants. Initial five categories were collapsed to three categories included in the logistic regression models.

#### Burden of chronic disease

Items included in the survey questionnaires related to the burden of chronic disease for the respondent included duration of chronic disease, hospitalizations resulting from its course, and number of chronic diseases.

#### Internet use

Respondents were asked about their Internet use and their opinions about the usefulness of the Internet in making decision about their own health. They could select one of three response options in relation to Internet use: independent use of the Internet, use of the Internet with help from others, and no use of the Internet at all. The item asking about the usefulness of the Internet (item 1 from the eHealth literacy scale according to Norman and Skinner) was assigned five response options: from not useful at all to very useful, with the neutral option in the middle [35].

#### Attitudes toward the use of the Internet for provision of services

The survey questionnaire included items asking about the acceptance of the use of specific eHealth applications. In this paper, the acceptance of the provision of the following online services was analysed: 1) accessing patient medical record, 2) making appointments to see physicians, 3) renewing prescriptions, 4) accessing results of laboratory tests, 5) accessing educational resources, and 6) consulting physicians. The relevant questionnaire items were formulated as “Do you think this service may be provided via the Internet?”, where the service means one of the six types of eHealth applications listed above. The responses to these items could be provided according to the five-point Likert scale from strongly agree to strongly disagree, with the neutral response in the middle position. Initial responses expressed with the five-point Likert scale were collapsed into two categories: ‘0’ – if the respondent selected “Strongly disagree”, “Rather disagree” or “Not sure/Don’t know”, and ‘1’ – if the respondent selected “Strongly agree” or “Rather agree”.

### Statistical analysis

Statistical analysis was performed using IBM SPSS v.21 (Armonk, NY, USA). Descriptive analysis was performed for the variables described in the paper. If not stated otherwise, the frequency of responses to specific items was given as a percentage of all valid responses excluding missing responses. The assessment of determinants of the acceptance of eHealth services was conducted with univariate and multivariate logistic regression models. The problem of missing data was addressed with the multiple imputation procedure. The p level below 0.05 was treated as significant.

### Missing values

Multiple imputation was performed on the initial data set to account for missing values, according to fully conditional specification procedure available in the SPSS v.21 package. All independent and dependent variables were included in the procedure [37]. The percentage of missing values was 9.9 %. The frequencies of missing values in the variables included in logistic regression models ranged from 0% to 22.9% (see Table 1). Given the high percentage of missing values in some variables, the imputation was repeated twenty times [38].

**Table 1:** **Frequencies of missing values**

**Variable**	**%**
Gender	0.3
Age	3.5
Place of residence	1.8
Education level	0.3
Number of chronic diseases diagnosed in the respondent	0
Duration of chronic disease	8.4
At least one admission to hospital due to chronic disease	0
Opinion about the usefulness of the Internet in helping to make decision about own health	12.7
Ability to use the Internet without help from other people	0
The acceptance of Internet use for accessing medical record	22.9
The acceptance of Internet use for making appointments to see a physician	19.2
The acceptance of Internet use for renewing prescriptions	20.5
The acceptance of Internet use for accessing results of laboratory tests	21.8
The acceptance of Internet use for accessing educational resources	21.52
The acceptance of Internet use for consulting physician	19.0

### Logistic regression modelling

The outcome variables were derived from items related to the acceptance of the provision of specific activities online. The predictors included in the model were gender, age, place of residence, education level, number of chronic diseases, duration of the chronic disease, hospitalization related to the chronic disease, Internet use, and opinion about the usefulness of the Internet for making personal health-related decisions. First, the influence of predictors on outcome variables was assessed with univariate logistic regression models. Then, the multivariate models were developed including all nine predictors used for the univariate models.

Multivariate logistic regression was preceded by multicollinearity diagnostic analysis with a calculation of variance inflation factor (VIF) values for independent variables. No concerns were raised, since all VIF values were below 2.0 (Table 2).

**Table 2:** **The results of multicollinearity tests for independent values**

**Independent variables**	**Dependent variables**					
	**The acceptance of internet use for accessing medical record**	**The acceptance of internet use for making appointment to see physician**	**The acceptance of internet use for renewing prescriptions**	**The acceptance of internet use for accessing results of laboratory tests**	**The acceptance of internet use for accessing educational resources**	**The acceptance of internet use for consulting physician**
	**VIF**	**VIF**	**VIF**	**VIF**	**VIF**	**VIF**
Gender	1.066	1.060	1.060	1.058	1.069	1.054
Age	1.615	1.570	1.615	1.623	1.630	1.580
Place of residence	1.205	1.217	1.210	1.209	1.205	1.191
Education	1.474	1.459	1.509	1.488	1.460	1.434
Number of chronic diseases	1.260	1.259	1.258	1.266	1.248	1.257
Duration of chronic diseases	1.072	1.075	1.079	1.072	1.061	1.078
Hospitalization due to chronic disease	1.058	1.049	1.051	1.055	1.061	1.050
Internet use	1.762	1.689	1.748	1.770	1.733	1.668
Opinion about usefulness of Internet	1.149	1.146	1.142	1.145	1.149	1.146

VIF – variance inflation factor.

Multivariate logistic regression was conducted by the forward method available in the SPSS v.21 package. The odds ratios (OR) and 95% confidence intervals (95%CI) were calculated for predictor variables both in univariate and multivariate logistic regression models. Sensitivity analysis was performed using a nonimputed data set for multiple regression modelling (see Additional file 2) for OR and 95%CI). The results were not contradictory in relation to the pooled results of logistic regression modelling on 20 imputed data sets. Statistical significance changed only in case of a few independent variables when pooled results of multiple logistic regressions carried out on multiply-imputed data set were compared to the results on nonimputed data set.

## Results

### Characteristics of respondents

Questionnaires were completed by 400 patients from 524 approached by canvassers asking them to join the survey (response rate 76.3%). Following quality checks, five questionnaires were excluded from the analysis because of missing sociodemographic data. Women made up 64.2% of the respondents. The mean age of the respondents (SD) was 47.9 (17.7) years, with 46.6 (17.9) years for women and 50.2 (17.4) years for men. The median age was 50 years, with 31 as the lower quartile and 61 as the higher quartile. The percentage of Internet users (respondents who access the Internet on their own without help from other people) was 60.3% in the study group. More than one chronic disease was diagnosed in 46.3% of respondents. At least one hospitalization due to chronic disease was reported by 71.4% of respondents. Median chronic disease duration was 10 years, with lower and upper quartiles at 5 and 18 years respectively. Detailed information on the frequencies of gender, age categories, level of education, place of residence of respondents, the variables showing the burden of disease, prevalence of chronic diseases, Internet use and opinion about the usefulness of the Internet for making decision about the patient’s own health is shown in Table 3.

**Table 3:** **Characteristics of respondents**

**Variable**		**n**	**%**
Gender	Female	253	64.2
	Male	141	35.8
Age of respondent	≤31	100	26.3
	>31 to ≤50	96	25.2
	>50 to ≤61	93	24.4
	>61	92	24.1
Place of residence	Rural	120	30.9
	Urban below 100.000 inhabitants	91	23.5
	Urban above 100.000 inhabitants	177	45.6
Education level	Level A	129	32.7
	Level B	124	31.5
	Level C	141	35.8
Number of chronic diseases diagnosed in the respondent	1	212	53.7
	>1	183	46.3
Prevalence of chronic diseases among respondents	Cardiovascular diseases apart from arterial hypertension	77	19.5
	Arterial hypertension	104	26.3
	Diabetes	153	38.7
	Bronchial asthma	79	20.0
	Chronic obstructive pulmonary disease	48	12.2
	Disease of musculoskeletal system	89	22.5
	Neurological disease	35	8.9
	Depression	38	9.6
	Other	100	25.3
Duration of chronic disease	≤5	93	25.7
	>5 to ≤10	103	28.5
	>10 to ≤18	78	21.5
	>18	88	24.3
At least one admission to hospital due to chronic disease	No	113	28.6
	Yes	282	71.4
Ability to use the Internet without help from other people	No	157	39.7
	Yes	238	60.3
Opinion about the usefulness of the Internet in helping to make decision about own health	Not useful at all	34	8.6
	Not useful	34	8.6
	Unsure	80	20.3
	Useful	150	38.0
	Very useful	47	11.9
The acceptance of Internet use for accessing medical record	No or not sure	123	39.3
	Yes	176	60.7
The acceptance of Internet use for making appointment to see physician	No or not sure	72	22.6
	Yes	247	77.4
The acceptance of Internet use for renewing prescriptions	No or not sure	125	39.8
	Yes	189	60.2
The acceptance of Internet use for accessing results of laboratory tests	No or not sure	112	36.3
	Yes	197	63.8
The acceptance of Internet use for accessing educational resources	No or not sure	155	48.4
	Yes	165	51.6
The acceptance of Internet use for consulting physician	No or not sure	103	33.1
	Yes	208	66.9

### Determinants of opinions about the use of the Internet for the provision of health care services

#### Accessing medical record

The frequency of responses accepting the use of the Internet for accessing patient medical record was 60.7%. Higher acceptance was related to younger age, inhabitance in urban areas, higher education level, only one chronic disease in respondent, no previous hospitalizations caused by the chronic disease, the use of the Internet, and a higher conviction that the Internet is useful for making personal health-related decisions. Respondents with the chronic disease lasting 5–10 years were less prone to accept Internet-based access that those with duration of disease not surpassing 5 years. OR and 95%CI for specific determinants are presented in Table 4. When combined in the multivariate logistic regression model (Table 4), a statistically significant effect was maintained by the level of education (highest education level in relation to reference level of education; OR 2.52; 95%CI 1.12-5.67), previous hospitalization due to chronic disease (no hospitalization vs. at least one hospitalization, OR 0.44, 95%CI 0.22-0.85), and opinion about the usefulness of the Internet for making personal health-related decisions (OR for comparison between the lowest conviction about the usefulness and higher levels; from 3.80 to 6.02 with no difference in relation to the middle response). The odds that respondents with the highest level of education will accept accessing medical documentation online were 2.52 times greater than for respondents from the group with the lowest level of education. The respondents who were highly convinced about the usefulness of the Internet in making health-related decision had 6 times higher odds that they will accept such access than those who believed that the Internet was not useful at all.

**Table 4:** **The results of univariate and multivariate logistic regression models of the acceptance of Internet use for accessing medical record**

**Independent variables**	**Unadjusted OR (95% CI)**	**p**	**Adjusted OR (95% CI)**	**p**
Gender				
Female				
Male	1.02 (0.66-0.92)	.92	1.27 (0.71-2.28)	.42
Age of respondent				
≤31 years				
>31 to ≤50 years	0.53 (0.27-1.01)	.05	0.73 (0.34-1.58)	.42
>50 to ≤61 years	0.32 (0.16-0.62)	.001	0.62 (0.26-1.48)	.28
>61 years	0.20 (0.10-0.39)	<.001	0.46 (0.18-1.19)	.11
Place of residence				
Rural				
Urban below 100.000 inhabitants	0.95 (0.53-1.72)	.87	0.67 (0.32-1.37)	.27
urban above 100.000 inhabitants	2.28 (1.31-3.95)	.004	1.77 (0.86-3.66)	.12
Education				
Level 1				
Level 2	2.14 (1.15-3.97)	.02	1.35 (0.62-2.97)	.45
Level 3	4.88 (2.72-7.75)	<.001	2.52 (1.12-5.67)	.03
Number of chronic diseases diagnosed in the respondent				
1				
>1	0.56 (0.36-0.86)	.009	0.92 (0.51-1.66)	.78
Duration of chronic disease				
≤5				
>5 to ≤10	0.51 (0.27-0.94)	.03	0.56 (0.27-1.16)	.12
>10 to ≤18	0.71 (1.36-1.38)	.31	0.84 (0.37-1.92)	.68
>18	0.55 (0.28-1.07)	.08	0.79 (0.34-1.80)	.57
At least one admission to hospital due to chronic disease				
No				
Yes	0.47 (0.27-0.81)	.007	0.44 (0.22-0.85)	.02
The use of the internet without help of other persons				
No				
Yes	4.12 (2.47-6.89)	<.001	1.61 (0.77-3.37)	.21
Opinion about the usefulness of the internet in helping to make decision about own health				
Not useful at all				
Not useful	4.21 (1.38-12.87)	.01	3.80 (1.16-12.42)	.03
Unsure	2.23 (0.84-5.92)	.11	1.70 (0.59-4.90)	.37
Useful	6.93 (2.64-18.19)	<.001	4.07 (1.41-11.72)	.01
Very useful	9.28 (3.00-27.71)	<.001	6.02 (1.78-20.32)	.004

#### Making appointment to see physician

Among services provided with eHealth, making appointments with physician received the highest acceptance from respondents (77.4%). The predictors which demonstrated a statistically significant influence on the acceptance in the univariate model included age, education, number of chronic diseases, duration of chronic disease, use of the Internet, and opinion about the usefulness of the Internet in making personal health-related decisions. In the multivariate logistic regression model, the only independent variable which maintained a significant influence was the opinion about Internet usefulness (OR and 95%CI for comparison between options “not useful at all” and “unsure”, “useful” and “very useful” were 3.56, 1.25-10.18; 6.94, 2.13-22.59 and 11.06, 2.49-49.01, respectively). The odds that respondents convinced about a high usefulness of the Internet for making health-related decisions have a positive opinion about the possibility of using the Internet for making appointment to see physician were 11 times higher when compared with respondents who believed that the Internet was not useful at all for making such decisions. The results of both models are presented in Table 5.

**Table 5:** **The results of univariate and multivariate logistic regression models for the acceptance of Internet use for making appointment to see physician**

**Independent variables**	**Unadjusted OR (95% CI)**	**p**	**Adjusted OR (95% CI)**	**p**
Gender				
Female				
Male	0.95 (0.54-1.68)	.87	0.97 (0.50-1.88)	.93
Age of respondent				
≤31 years				
>31 to ≤50 years	0.70 (0.32-1.49)	.35	1.10 (0.46-2.2)	.83
>50 to ≤61 years	0.53 (0.25-1.11)	.09	1.24 (0.47-3.26)	.66
>61 years	0.34 (0.15-0.74)	.007	1.12 (0.33-3.76)	.85
Place of residence				
Rural				
Urban below 100.000 inhabitants	1.14 (0.60-2.18)	.68	0.66 (0.31-1.44)	.30
Urban above 100.000 inhabitants	1.68 (0.92-3.06)	.09	1.24 (0.59-2.61)	.57
Education				
Level 1				
Level 2	1.56 (0.81-3.03)	.18	.814	.63
Level 3	3.30 (1.69-6.44)	<.001	1.707	.24
Number of chronic diseases diagnosed in the respondent				
1				
>1	0.55 (0.33-0.91)	.02	0.68 (0.35-1.32)	.25
Duration of chronic disease				
≤5				
>5 to ≤10	0.42 (0.19-0.93)	.03	0.46 (0.20-1.09)	.08
>10 to ≤18	0.60 (0.25-1.42)	.24	0.66 (.24-1.81)	.42
>18	0.65 (0.27-1.55)	.33	0.84 (0.31-2.31)	.73
At least one admission to hospital due to chronic disease				
No				
Yes	0.60 (0.31-1.19)	.14	0.67 (0.31-1.44)	.30
The use of the internet without help of other persons				
No				
Yes	3.07 (1.74-5.39)	<.001	1.51 (0.68-3.34)	.31
Opinion about the usefulness of the internet in helping to make decision about own health				
Not useful at all				
Not useful	2.65 (0.81-8.64)	.11	2.57 (0.70-9.38)	.15
Unsure	3.48 (1.34-8.91)	.009	3.56 (1.25-10.18)	.02
Useful	7.74 (2.93-20.46)	<.001	6.94 (2.13-22.60)	.002
Very useful	12.80 (3.31-49.57)	<.001	11.06 (2.49-49.01)	.002

#### Renewing prescriptions

The acceptance of eHealth application for renewing prescriptions reached 60.2%. The variable which predicted the acceptance in univariate logistic regression models included age, place of residence, education, duration of chronic disease, use of the Internet, and the opinion about the usefulness of the Internet in making personal health-related decisions. The only predictor in the multivariate model was the use of the Internet (OR and 95%CI for comparison of users with non-users 3.23, 1.56-6.69). This means that Internet users were 3.23 times more likely to accept renewing prescriptions online than nonusers. Detailed results of both univariate and multivariate logistic regression models are presented in Table 6.

**Table 6:** **The results of univariate and multivariate logistic regression models for the acceptance of Internet use for renewing prescriptions**

**Independent variables**	**Unadjusted OR (95% CI)**	**p**	**Adjusted OR (95% CI)**	**p**
Gender				
Female				
Male	0.87 (0.55-1.38)	.55	0.83 (0.47-1.47)	.51
Age of respondent				
≤31 years				
>31 to ≤50 years	0.51 (0.27-0.94)	.03	0.70 (0.35-1.39)	.31
>50 to ≤61 years	0.54 (0.29-1.00)	.05	1.09 (0.49-2.42)	.84
>61 years	0.39 (0.20-0.75)	.005	1.11 (0.42-2.95)	.84
Place of residence				
Rural				
Urban below 100.000 inhabitants	1.27 (0.71-2.29)	.43	0.85 (0.43-1.67)	.63
Urban above 100.000 inhabitants	2.58 (1.45-4.56)	.001	1.81 (0.90-3.61)	.09
Education				
Level 1				
Level 2	1.97 (1.10-3.50)	.02	1.01 (0.49-2.12)	.97
Level 3	4.41 (2.52-7.72)	<.001	1.74 (0.84-3.61)	.14
Number of chronic diseases diagnosed in the respondent				
1				
>1	0.73 (0.48-1.12)	.18	0.92 (0.53-1.58)	.75
Duration of chronic disease				
≤5				
>5 to ≤10	0.51 (0.27-0.96)	.04	0.58 (0.29-1.19)	.14
>10 to ≤18	0.82 (0.40-1.66)	.57	0.99 (0.43-2.28)	.98
>18	0.82 (0.43-1.56)	.54	1.07 (0.50-2.29)	.87
At least one admission to hospital due to chronic disease				
No				
Yes	0.66 (0.39-1.11)	.12	0.59 (0.32-1.09)	.09
The use of the internet without help of other persons				
No				
Yes	4.19 (2.53-6.96)	<.001	3.23 (1.56-6.69)	.002
Opinion about the usefulness of the Internet in helping to make decision about own health				
Not useful at all				
Not useful	2.02 (0.68-6.02)	.21	1.95 (0.58-6.53)	.28
Unsure	1.72 (0.67-4.39)	.26	1.41 (0.49-4.10)	.52
Useful	2.89 (1.22-6.87)	.02	1.60 (0.60-4.26)	.34
Very useful	3.18 (1.11-9.09)	.03	1.82 (0.56-5.90)	.32

#### Accessing results of laboratory tests

The acceptance of using the Internet for accessing laboratory test results was 63.8%. The predictors of the acceptance in univariate logistic regression models included age, place of residence, education, admission to hospital due to chronic disease, the use of the Internet, and the opinion about the usefulness of the Internet in making personal health-related decisions. In the multivariate model, a statistically significant impact was maintained by education (OR and 95%CI for comparison between the lowest and the highest levels of education: 2.56 and 1.12-5.86, respectively), admission to hospital due to chronic disease (OR and 95%CI for comparison between no admission and at least one admission: 0.52 and 0.27-0.98, respectively) and the opinion about the usefulness of the Internet in making health-related decisions (OR and 95% for comparison between response option “not useful at all” and more favourable opinions about the usefulness: 4.43, 1.21-16.29; 3.26, 1.09-9.79; 6.64, 2.28-19.30 and 4.81, 1.45-15.96, respectively). The results show that respondents, who have been admitted to hospital due to chronic disease at least once, were 0.52 times less likely to accept accessing results of laboratory tests results online than respondents who have not been hospitalized. Respondents with the highest education level showed 2.56 higher odds of accepting online provision of this service when compared to individuals with the lowest education level. Detailed results of both models are included in Table 7.

**Table 7:** **The results of univariate and multivariate logistic regression models for the acceptance of Internet use for accessing results of laboratory tests**

**Independent variables**	**Unadjusted OR (95% CI)**	**p**	**Adjusted OR (95% CI)**	**p**
Gender				
Female				
Male	0.93 (0.57-1.51)	.76	1.04 (0.57-1.90)	.90
Age of respondent				
≤31 years				
>31 to ≤50 years	1.06 (0.54-2.06)	.87	1.64 (0.75-3.61)	.21
>50 to ≤61 years	0.39 (0.20-0.73)	.003	0.70 (0.30-1.63)	.41
>61 years	0.34 (0.17-0.66)	.002	0.85 (0.31-2.36)	.75
Place of residence				
Rural				
Urban below 100.000 inhabitants	1.20 (0.65-2.21)	.56	0.87 (0.42-1.80)	.70
Urban above 100.000 inhabitants	2.42 (1.37-4.27)	.002	1.95 (0.91-4.17)	.09
Education				
Level 1				
Level 2	1.73 (0.96-3.14)	.07	1.02 (0.47-2.23)	.96
Level 3	4.79 (2.59-8.86)	<.001	2.56 (1.12-5.86)	.03
Number of chronic diseases diagnosed in the respondent				
1				
>1	0.65 (0.41-1.01)	.06	0.90 (0.49-1.67)	.74
Duration of chronic disease				
≤5				
>5 to ≤10	0.76 (0.40-1.45)	.41	0.89 (0.41-3.85)	.76
>10 to ≤18	1.18 (0.57-2.47)	.65	1.53 (0.61-3.86)	.36
>18	1.08 (0.54-2.19)	.83	1.64 (0.66-4.10)	.28
At least one admission to hospital due to chronic disease				
No				
Yes	0.53 (0.31-0.91)	.02	0.52 (0.27-0.98)	.04
The use of the internet without help of other persons				
No				
Yes	3.68 (2.23-6.08)	<.001	1.65 (0.79-3.42)	.18
Opinion about the usefulness of the internet in helping to make decision about own health				
Not useful at all				
Not useful	4.54 (1.37-15.06)	.01	4.43 (1.21-16.29)	.03
Unsure	3.44 (1.21-9.83)	.02	3.26 (1.09-9.79)	.04
Useful	9.33 (3.50-24.89)	<.001	6.64 (2.28-19.30)	.001
Very useful	6.76 (2.25-20.33)	<.001	4.81 (1.45-15.96)	.01

#### Accessing educational resources for patients

Online access to education resources for patients was accepted by 66.9% respondents. Independent variables predicting the acceptance of this application in univariate logistic regression models were education, place of residence, education, duration of chronic disease, hospitalization due to chronic disease, the use of the Internet and the opinion about the usefulness of the Internet in making personal health-related decisions. In the multivariate model, a statistically significant impact was maintained by education (OR and 95%CI for comparison between the highest and lowest levels of education: 2.51, 1.09-5.74), hospitalization due to chronic disease (OR and 95%CI for comparison between no admission and at least one admission to hospital due to chronic disease: 0.51, 0.27-0.96), duration of chronic disease (OR and 95%CI for comparison between respondents with the disease lasting ≤5 and those with the disease lasting >5 to 10 years: 0.30, 0.14-0.64) and the opinion about the usefulness of the Internet in making personal health-related decisions. For the last independent variable, OR and 95%CI for comparison between the response option “not useful at all” and options “not useful”, “useful” and “very useful” were 4.17, 1.15-14.21; 7.56, 2.29-24.95, and 11.38, 2.73-47.47, respectively. All results of logistic regression modelling in relation to accessing education resources online as an independent variable are shown in Table 8.

**Table 8:** **The results of univariate and multivariate logistic regression models for the acceptance of Internet use for accessing educational resources**

**Independent variables**	**Unadjusted OR (95% CI)**	**p**	**Adjusted OR (95% CI)**	**p**
Gender				
Female				
Male	0.91 (0.57-1.44)	.68	1.01 (0.56-1.82)	.98
Age of respondent				
≤31 years				
>31 to ≤50 years	0.59 (0.29-1.18)	.132	0.88 (0.39-2.00)	
>50 to ≤61 years	0.37 (0.18-0.74)	.005	0.77 (0.29-2.04)	.60
>61 years	0.26 (0.14-0.51)	<.001	0.79 (0.28-2.23)	.66
Place of residence				
rural				
urban below 100.000 inhabitants	1.12 (0.59-2.11)	.73	0.67 (0.29-1.51)	.33
urban above 100.000 inhabitants	1.89 (1.10-3.27)	.02	1.40 (0.68-2.88)	.36
Education				
level 1				
level 2	2.404	.005	1.47 (0.66-3.26)	.35
level 3	4.527	<.001	2.51 (1.09-5.74)	.03
Number of chronic diseases diagnosed in the respondent				
1				
>1	0.50 (0.32-0.80)	.004	0.69 (0.36-1.33)	.27
Duration of chronic disease				
≤5				
>5 to ≤10	0.31 (0.16-0.59)	<.001	0.30 (0.14-0.64)	.002
>10 to ≤18	0.56 (0.270-1.18)	.13	0.60 (0.24-1.49)	.27
>18	0.54 (0.26-1.12)	.10	0.72 (0.31-1.65)	.43
At least one admission to hospital due to chronic disease				
No				
Yes	0.50 (0.30-0.84)	.009	0.51 (0.27-.96)	.04
The use of the internet without help of other persons				
No				
Yes	3.60 (2.15-6.04)	<.001	1.35 (0.60-3.03)	.472
Opinion about the usefulness of the internet in helping to make decision about own health				
Not useful at all				
Not useful	4.17 (1.23-14.14)	.02	4.17 (1.15-15.21)	.03
Unsure	3.50 (1.20-10.17)	.02	3.24 (0.98-10.76)	.06
Useful	9.79 (3.49-27.46)	<.001	7.56 (2.29-24.95)	.001
Very useful	15.25 (4.26-54.68)	<.001	11.38 (2.73-47.47)	.001

#### Consulting physician

The acceptance of using the Internet for consulting physician was the lowest among independent variables (51.6%). Independent variables with a statistical significance in univariate and multivariate logistic regression models were education, duration of chronic disease and the opinion about the usefulness of the Internet in making personal health-related decisions (Table 9). The values of OR and 95%CI for comparison between respondents with the lowest and highest level of education were 2.33 and 1.31-4.12, respectively. As for duration of the chronic disease, the only significant difference was between respondents with the chronic disease lasting not longer than 5 years and those with the disease lasting more than 10 years and not longer than 18 years (OR and 95%CI: 2.48, 1.22-5.03). This means that the odds of accepting online consultations with physicians by patients with the shortest duration of chronic disease were 2.48 times lower than the odds of patients with the diseases lasting more than 10 years and not longer than 18 years. However, higher approval levels for this type of online service were not seen among respondents suffering from chronic diseases for longer periods. As for the opinion about the usefulness of the Internet for making personal health-related decisions, the differences were statistically significant for comparison between the option “not useful at all” and options “useful” and “very useful” (OR and 95%CI for these comparisons: 3.39, 1.34-8.57 and 4.60, 1.51-13.98, respectively).

**Table 9:** **The results of univariate and multivariate logistic regression models for the acceptance of Internet use for consulting physician**

**Independent variables**	**Unadjusted OR (95% CI)**	**p**	**Adjusted OR (95% CI)**	**p**
Gender				
Female				
Male	0.87 (0.55-1.39)	.57	1.02 (0.60-1.74)	.95
Age of respondent				
≤31 years				
>31 to ≤50 years	0.98 (0.54-1.76)	.94	1.31 (0.67-2.54)	.44
>50 to ≤61 years	0.90 (0.50-1.62)	.72	1.71 (0.80-3.68)	.17
>61 years	1.04 (0.53-2.02)	.92	2.38 (0.96-5.87)	.06
Place of residence				
Rural				
Urban below 100.000 inhabitants	0.94 (0.52-1.70)	.84	0.66 (0.33-1.30)	.22
Urban above 100.000 inhabitants	1.08 (0.64-1.80)	.78	0.78 (0.41-1.49)	.46
Education				
Level 1				
Level 2	1.41 (0.80-2.50)	.24	1.30 (0.65-2.60)	.45
Level 3	2.33 (1.31-4.12)	.004	2.54 (1.21-5.33)	.01
Number of chronic diseases diagnosed in the respondent				
1				
>1	0.78 (0.50-1.21)	.26	0.66 (0.37-1.16)	.15
Duration of chronic disease				
≤5				
>5 to ≤10	0.79 (0.42-1.49)	.47	0.92 (0.47-1.79)	.81
>10 to ≤18	2.48 (1.22-5.03)	.01	2.96 (1.37-6.42)	.006
>18	1.39 (0.74-2.60)	.31	1.55 (0.77-3.14)	.22
At least one admission to hospital due to chronic disease				
No				
Yes	0.79 (0.49-1.26)	.32	0.77 (0.46-1.31)	.34
The use of the internet without help of other persons				
No				
Yes	1.33 (0.81-2.19)	.26	0.87 (0.44-1.73)	.70
Opinion about the usefulness of the internet in helping to make decision about own health				
Not useful at all				
Not useful	2.10 (0.75-5.84)	.16	2.61 (0.83-8.26)	.10
Unsure	1.14 (0.44-2.94)	.79	1.28 (0.46-3.60)	.63
Useful	2.78 (1.22-6.120	.01	3.39 (1.34-8.57)	.01
Very useful	3.45 (1.29-9.24)	.01	4.60 (1.51-13.98)	.007

## Discussion

### Principal results

Poland’s accession to the European Union stimulated activities focused on the development of information systems supporting public administration, and the provision of services to citizens, including health care applications. Target areas for development in relation to health care services encompass solutions including patient medical records accessible online, applications for booking appointments with physicians, e-prescribing system, and applications providing access to laboratory test results [39,40]. Using information systems for interactions between patients and health care providers and enhancing the support of patients with access to educational resources was also considered. Implementing eHealth solutions involving patients as end-users requires careful planning and anticipating potential reactions and compliance. This study assessed the acceptance of specific eHealth solutions forming priority areas for national strategies among patients with chronic diseases.

Results of univariate models applied to six types of eHealth solutions consistently revealed that sociodemographic variables had a significant impact on their acceptance. Most eHealth solutions were more accepted by respondents of younger age, higher education level, and urban place of residence with above 100,000 inhabitants. Furthermore, patients using the Internet on their own, and expressing a positive opinion about the usefulness of the Internet in making personal health-related decisions were more likely to accept most of the eHealth solutions included in the analysis. These finding are in line with results of studies performed in various patient groups in other countries [41-51]. The same determinants were also reported for health-related Internet use or acceptance in general populations [52-56]. It is worth noting that our study did not reveal an effect of gender on the acceptance of eHealth systems. Many other studies have reported higher health-related Internet use among women [53,55-59].

Variables related to the burden of disease (number of chronic diseases, duration of chronic disease and hospitalization due to chronic disease) also showed a significant effect on the acceptance of specific applications. Hospitalization due to chronic disease and multiple chronic diseases diagnosed in a patient were both associated with a lower acceptance of three of the six eHealth applications included in the logistic regression models. As for the duration of chronic disease, the acceptance of four of the six eHealth applications was significantly lower among patients with the disease lasting from above 5 to 10 years in comparison to patients with disease duration ≤5 years. Interestingly, the comparison between patients with the shortest disease duration and two categories with the longest duration did not show statistically significant differences. The use of the Internet for consulting physician was more accepted by patients with disease duration from above 10 to 18 years in comparison with the category with the shortest disease duration. There have been some studies indicating a lower use or acceptance of using the Internet for health-related purposes among respondents with a higher disease burden [60,61]. A study on data from a nationwide survey of households in Poland revealed that the hospitalization of a member of a household did not have a significant impact on the acceptance of Internet-based health care services, and recent use of health care services was related only to the acceptance of a limited form of health service online [62]. Other studies have reported a higher acceptance or use of the Internet for health-related purposes among people with previous or existing medical conditions [57,58,63-65].

It should be noted that a longer duration of disease or more than one chronic condition occurring in the same patient are frequently correlated to his or her older age, which in turn is generally associated with lower Internet use, also in the context of health care services.

When included in the multivariate model, the significant effect on the acceptance of specific eHealth solutions was maintained consistently by variables indicating the respondent’s opinion on the usefulness of the Internet for making decisions on personal health, and their education level. The variable related to the respondent’s view on the usefulness of the Internet had a significant impact on all but one independent variable. Education level remained a significant predictor of the acceptance of four types of eHealth solutions analysed except making appointment to see physician and renewing prescriptions. From other independent variables, the hospitalization due to the chronic disease was related to lower acceptance of types of eHealth services and Internet use to higher acceptance of renewing prescriptions online. The impact of duration of the chronic disease was observed in two types of eHealth services.

Our results suggest that the respondents’ views on the usefulness of the Internet for making personal health-related decisions could serve as a universal predictor of patient attitudes towards the implementation and use of eHealth solutions. In five out of six multivariate logistic regression models, it maintained its effect on the acceptance of eHealth solutions. In relation to the acceptance of making appointment with physician online, it appeared to be the only predictor.

In the multivariate logistic regression models, the independent effects of age and place of residence on the acceptance vanished. This is likely due to the fact that the opinion about the usefulness of the Internet for personal health combines the effects stemming from the respondent’s sociodemographic background, especially age and place of residence.

E-prescription was the only application with the acceptance significantly influenced by using the Internet rather than by a positive opinion about the usefulness of the Internet for making personal health-related decisions. For the remaining four applications, the effect of Internet use was overridden by the opinion about its usefulness. This finding may serve as an important indication that Internet use as such is not a sufficient determinant of the acceptance of eHealth services.

### Limitations

The study suffered from several limitations. First of all, the group of respondents was limited to patients obtaining care from selected health care facilities in one large city located in southern Poland. Thus, broader extrapolation of the results of the study should be made with caution. On the other hand, the percentage of patients living in rural areas was sufficiently high to provide feedback on their opinions. Two of the three medical departments providing care to respondents serve as referral centres in their specialty areas, which could have an impact on both the characteristics of the patients and their perceived needs for additional or alternative ways of support including eHealth. Another weakness was the high frequency of missing values, especially in relation to variables related to the acceptance of specific forms of eHealth services. To remedy this, the multiple imputation procedure was employed. The comparison of the results of multiple regression modelling performed on the non-imputed data set and pooled results of the analysis on data sets resulting from multiple imputation did not show essential differences. There were only a few discrepancies in terms of predictors having a statistically significant impact on independent variables. Finally, respondents included in the survey should be treated as an eHealth-naive population. Most patients in Poland have only a limited access to advanced eHealth systems. Their opinions about the feasibility of eHealth applications do not come from direct experience, but are likely influenced mainly by their experience of general Internet use, also in health-related context, and the use of electronic public administration applications in some cases.

## Conclusions

The study demonstrates that the acceptance of specific eHealth solutions among patients with chronic conditions depends on their general attitude toward the usefulness of the Internet for personal health. Two other important predictors of the acceptance included the patient’s education and previous hospitalizations due to the chronic disease. The effects of sociodemographic factors such as age or place of residence as well as Internet use were not maintained as statistically significant predictors in multivariate models. These observations may lead to the conclusion that implementation of eHealth applications addressed to chronic patients should be preceded with interventions focused on increasing their understanding and acceptance of the eHealth domain. Without appropriate preparation of target audiences, the group of active users may be limited to younger, better educated patients from highly populated urban areas. Furthermore, the use of the Internet is in itself not sufficient to improve the acceptance of eHealth solutions.

## Additional files

Additional file 1:
**Questionnaire items used for the analysis presented in the paper (English and Polish versions).**
Additional file 2:
**The results of multiple logistic regression modelling on nonimputed data set.**
