# Supplementary material for: A cross-sectional study assessing determinants of the attitude to the introduction of eHealth services among patients suffering from chronic conditions
Source: BMC Med Inform Decis Mak. 2015 Apr 19;15:33. doi: 10.1186/s12911-015-0157-3 (PMC4409745; doi:10.1186/s12911-015-0157-3)
Supplement: Additional file 1: — Questionnaire items used for the analysis presented in the paper (English and Polish versions). [file 12911_2015_157_MOESM1_ESM.doc]

Additional File 1. Questionnaire items used for the analysis presented in the paper

**English version**

**I.1 Do you suffer from chronic disease?**

1. cardiovascular disease 
2. arterial hypertension 
3. diabetes 
4. bronchial asthma 
5. chronic pulmonary obstructive disease 
6. skeletal disease 
7. neurological disease 
8. depression 
9. other disease 

if yes, please specify what disease? ……….............................................................

**I.2. How many years do you suffer from chronic disease?** …………………….....................

**I.3. Were you admitted to the hospital due to course of the disease??**

1. yes 
2. no 

**III. 4 Do you use the Internet?**

1. yes, on my own 
2. yes, but with help of other persons 
3. no 

**IV.1. What is your opinion about the usefulness of the Internet in helping to make decision about own health?**

- 1. not useful at all 
  2. not useful 
  3. unsure 
  4. useful 
  5. very useful 

**V.12. Do you think the services listed below may be provided via the Internet?**

|  | strongly agree | agree | neither agree nor disagree | disagree | strongly agree |
| --- | --- | --- | --- | --- | --- |
| consulting physician |  |  |  |  |  |
| making appointment online to see physician |  |  |  |  |  |
| accessing medical record |  |  |  |  |  |
| accessing educational resources |  |  |  |  |  |
| accessing results of laboratory tests |  |  |  |  |  |
| renewing prescriptions |  |  |  |  |  |

**VI.1 What is your age (in years)? ………….**

**VI. 2 What is your gender?**

- 1. male 
  2. female 

**VI. 3 What is your place of residence?**

1. urban > 500 000 inhabitants 
2. urban 100000 – 500000 inhabitants 
3. urban 10 000 – 100000 inhabitants 
4. urban <10 000 inhabitants 
5. rural 

**VI.4 What is your education?**

1. primary (podstawowe) 
2. lower secondary (gimnazjum) 
3. secondary vocational (zasadnicze zawodowe) 
4. not full secondary (niepełne średnie) 
5. secondary (średnie) 
6. post secondary non-tertiary (pomaturalne) 
7. first stage of tertiary (licencjat) 
8. not full tertiary (niepełne wyższe) 
9. tertiary (wyższe) 

**Questionnaire items used for the analysis presented in the paper ( Polish version).**

**I.1 Czy choruje Pan na chorobę przewlekłą?**

1. choroby układu krążenia 
2. nadciśnienie tętnicze 
3. cukrzyca 
4. astma oskrzelowa 
5. przewlekła obturacyjna choroba płuc 
6. choroba układu kostno-stawowego 
7. choroba układu nerwowego 
8. depresja 
9. inne schorzenie 

**jeśli na inną, proszę podać na jaką?** ……….............................................................

**I.2. Od ilu lat choruje Pan/i na chorobę przewlekłą?**……………………..

**I.3. Czy był Pan/i hospitalizowany z powodu zaostrzenia dolegliwości tej choroby?**

1. tak 
2. nie 

**III. 4 Czy korzysta Pan/Pani z Internetu?**

1. tak, samodzielnie 
2. tak, za pośrednictwem innych osób 
3. nie 

**IV.1. Jakie są Pana/i odczucia co do przydatności Internetu w podejmowaniu decyzji dotyczących Twojego zdrowia?**

1. zupełnie nieprzydatny 
2. nieprzydatny 
3. nie jestem pewien 
4. przydatny 
5. bardzo przydatny 

V.12. Które z wymienionych poniżej czynności można według Pana/i realizować za pośrednictwem Internetu?

|  | zdecydo-wanie tak | raczej tak | nie jestem pewny/a; nie wiem | raczej nie | zdecydo-wanie nie |
| --- | --- | --- | --- | --- | --- |
| konsultacje z lekarzem |  |  |  |  |  |
| rejestracja na wizytę w gabinecie lekarskim/poradni |  |  |  |  |  |
| gromadzenie wyników badań i dokumentacji medycznej na osobistym koncie internetowym dostępnym z każdego miejsca |  |  |  |  |  |
| dostęp do zasobów edukacyjnych dostosowanych do potrzeb pacjenta |  |  |  |  |  |
| możliwość kontaktu z pracownikiem ochrony zdrowia w razie zaostrzenia dolegliwości choroby |  |  |  |  |  |
| dostęp do wyników badań oznaczonych w laboratorium diagnostycznym |  |  |  |  |  |
| zgłoszenie zapotrzebowania na odnowienie recepty do lekarza prowadzącego |  |  |  |  |  |

**VI.1 Pana/i wiek w latach: ………….**

**VI. 2 Pana/i płeć:**

1. mężczyzna 
2. kobieta 

**VI. 3 W jakiej miejscowości Pan/Pani mieszka?**

1. miasto > 500 000 mieszkańców 
2. miasto 100000 – 500000 mieszkańców 
3. miasto 10 000 – 100000 mieszkańców 
4. miasto <10 000 mieszkańców 
5. wieś 

**VI.4 Jakie jest Pana/i wykształcenie:**

1. podstawowe 
2. gimnazjum 
3. zasadnicze zawodowe 
4. niepełne średnie 
5. średnie 
6. pomaturalne 
7. licencjat 
8. niepełne wyższe 
9. wyższe 
