# Supplementary material for: A cross-sectional study assessing determinants of the attitude to the introduction of eHealth services among patients suffering from chronic conditions
Source: BMC Med Inform Decis Mak. 2015 Apr 19;15:33. doi: 10.1186/s12911-015-0157-3 (PMC4409745; doi:10.1186/s12911-015-0157-3)
Supplement: Additional file 2: — The results of multiple logistic regression modelling on nonimputed data set. [file 12911_2015_157_MOESM2_ESM.doc]

**Additional File 2. The results of multiple logistic regression modeling on nonimputed data set.**

## Table 1. Multiple logistic regression models for the acceptance of Internet use for accessing medical record and making appointment to see physician

| Independent variable | The acceptance of Internet use for accessing medical record | | The acceptance of Internet use for making appointment to see physician | |
| --- | --- | --- | --- | --- |
|  | Adjusted OR  (95% CI) | p | Adjusted OR  (95% CI) | p |
| Gender |  |  |  |  |
| Female |  |  |  |  |
| Male | 1.47 (0.77-2.81) | .25 | 0.95 (0.45-2.02) | .89 |
|  |  |  |  |  |
| Age of respondent |  |  |  |  |
| ≤31 years |  |  |  |  |
| >31 to ≤50 years | 0.75 (0.33-1.72) | .50 | 1.21 (0.46-3.18) | .70 |
| >50 to ≤61 years | 0.75 (0.30-1.88) | .54 | 1.60 (0.53-4.82) | .40 |
| >61 years | 0.54 (0.19-1.49) | .23 | 1.06 (0.31-3.59) | .92 |
| Place of residence |  |  |  |  |
| rural |  |  |  |  |
| urban below 100.000 inhabitants | 0.96 (0.42-2.18) | .92 | 0.85 (0.34-2.18) | .74 |
| urban above 100.000 inhabitants | 1.87 (0.89-3.93) | .10 | 1.51 (0.65-3.51) | .34 |
| Education |  |  |  |  |
| level 1 |  |  |  |  |
| level 2 | 1.45 (0.65-3.24) | .37 | 0.91 (0.37-2.26) | .84 |
| level 3 | 2.79 (1.190-6.55) | .02 | 1.62 (0.60-4.38) | .35 |
| Number of chronic diseases diagnosed in the respondent |  |  |  |  |
| 1 |  |  |  |  |
| >1 | 0.65 (0.34-1.23) | .18 | 0.51 (0.24-1.09) | .08 |
| Duration of chronic disease |  |  |  |  |
| ≤5 |  |  |  |  |
| >5 to ≤10 | 0.70 (0.32-1.54) | .37 | 0.55 (0.22-1.41) | .21 |
| >10 to ≤18 | 0.86 (0.37-2.00) | .72 | 0.70 (0.25-1.97) | .50 |
| >18 | 0.80 (0.35-1.84) | .60 | 0.71 (0.26-1.93) | .50 |
| At least one admission to hospital due to chronic disease |  |  |  |  |
| No |  |  |  |  |
| Yes | 0.26 (0.12-0.56) | 001 | 0.46 (0.20-1.10) | .08 |
| The use of the Internet without help of other persons |  |  |  |  |
| No |  |  |  |  |
| Yes | 1.18 (0.52-2.66) | .69 | 1.18 (0.47-2.92) | .73 |
| Opinion about the usefulness of the Internet in helping to make decision about own health |  |  |  |  |
| Not useful at all |  |  |  |  |
| Not useful | 2.53 (0.65-9.83) | .18 | 2.57 (0.69-9.53) | .16 |
| Unsure | 1.18 (0.36-3.83) | .79 | 4.92 (1.50-16.22) | .009 |
| Useful | 3.95 (1.24-12.55) | .02 | 10.85 (3.28-35.90) | .000 |
| Very useful | 4.38 (1.18-16.27) | .03 | 12.93 (2.92-57.37) | .001 |

**Table 2. Multiple logistic regression models for the acceptance of Internet use for renewing prescriptions and accessing results of laboratory tests as independent variables**

| Variable | The acceptance of Internet use for renewing prescriptions | | The acceptance of Internet use for accessing results of laboratory tests | |
| --- | --- | --- | --- | --- |
|  | Adjusted OR  (95% CI) | p | Adjusted OR  (95% CI) | p |
| Gender |  |  |  |  |
| Female |  |  |  |  |
| Male | 0.88 (0.49-1.60) | .68 | 1.21 (0.62-2.36) | .58 |
|  |  |  |  |  |
| Age of respondent |  |  |  |  |
| ≤31 years | 0.70 (0.33-1.47) | .34 | 1.50 (0.62-3.60) | .37 |
| >31 to ≤50 years | 1.14 (0.47-2.75) | .77 | 0.65 (0.25-1.65) | .36 |
| >50 to ≤61 years | 0.97 (0.36-2.61) | .95 | 0.66 (0.23-1.90) | .45 |
| >61 years |  |  |  |  |
| Place of residence |  |  |  |  |
| rural |  |  |  |  |
| urban below 100.000 inhabitants | 1.00 (0.47-2.13) | .99 | 1.04 (0.45-2.37) | .93 |
| urban above 100.000 inhabitants | 1.90 (0.95-3.78) | .07 | 2.28 (1.06-4.90) | .04 |
| Education |  |  |  |  |
| level 1 |  |  |  |  |
| level 2 | 1.50 (0.70-3.20) | .30 | 1.64 (0.73-3.72) | .23 |
| level 3 | 2.25 (1.01-5.03) | .048 | 3.75 (1.54-9.13) | .004 |
| Number of chronic diseases diagnosed in the respondent |  |  |  |  |
| 1 |  |  |  |  |
| >1 | 0.69 (0.38-1.28) | .24 | 0.71 (0.36-1.38) | .31 |
| Duration of chronic disease |  |  |  |  |
| ≤5 |  |  |  |  |
| >5 to ≤10 | 0.68 (0.33-1.40) | .29 | 1.09 (0.50-2.40) | .83 |
| >10 to ≤18 | 0.96 (0.44-2.13) | .93 | 1.45 (0.61-3.43) | .41 |
| >18 | 1.03 (0.47-2.28) | .94 | 1.42 (0.59-3.42) | .43 |
| At least one admission to hospital due to chronic disease |  |  |  |  |
| No |  |  |  |  |
| Yes | 0.44 (0.23-0.86) | .02 | 0.29 (0.14-0.64) | .002 |
| The use of the Internet without help of other persons |  |  |  |  |
| No |  |  |  |  |
| Yes | 2.12 (0.96-4.67) | .06 | 1.01 (0.44-2.32) | .98 |
| Opinion about the usefulness of the Internet in helping to make decision about own health |  |  |  |  |
| Not useful at all |  |  |  |  |
| Not useful | 2.26 (0.60-8.56) | .23 | 3.66 (0.90-15.00) | .07 |
| Unsure | 1.59 (0.51-4.98) | .43 | 2.97 (0.86-10.19) | .08 |
| Useful | 2.14 (0.71-6.46) | .18 | 7.40 (2.17-24.21) | .001 |
| Very useful | 1.91 (0.56-6.55) | .30 | 4.45 (1.17-17.00) | .03 |

**Table 3. Multiple logistic regression models for the acceptance of Internet use for accessing education resources and consulting physician as independent variables**

| Variable | | The acceptance of Internet use for accessing educational resources | | The acceptance of Internet use for consulting physician | |
| --- | --- | --- | --- | --- | --- |
|  | Adjusted OR  (95% CI) | | p | Adjusted  OR (95% CI) | p |
| Gender |  | |  |  |  |
| Female |  | |  |  |  |
| Male | 1.31 (0.66-2.60) | | .43 | 1.10 (0.64-1.92) | .73 |
|  |  | |  |  |  |
| Age of respondent |  | |  |  |  |
| ≤31 years |  | |  |  |  |
| >31 to ≤50 years | 1.04 (0.44-2.46) | | .94 | 1.25 (0.62-2.51) | .54 |
| >50 to ≤61 years | 1.14 (0.43-3.04) | | .80 | 1.71 (0.77-3.78) | .19 |
| >61 years | 0.87 (0.29-2.62) | | .80 | 2.19 (0.87-5.52) | .10 |
| Place of residence |  | |  |  |  |
| rural |  | |  |  |  |
| urban below 100.000 inhabitants | 0.68 (0.29-1.60) | | .38 | 0.87 (0.42-1.83) | .71 |
| urban above 100.000 inhabitants | 1.29 (0.59-2.81) | | .52 | 0.85 (0.44-1.64) | .64 |
| Education |  | |  |  |  |
| level 1 |  | |  |  |  |
| level 2 | 1.88 (0.83-4.26) | | .13 | 1.22 (0.58-2.57) | .59 |
| level 3 | 2.95 (1.22-7.13) | | .02 | 2.23 (1.03-4.84) | .04 |
| Number of chronic diseases diagnosed in the respondent |  | |  |  |  |
| 1 |  | |  |  |  |
| >1 | 0.52 (0.27-1.03) | | .06 | 0.68 (0.39-1.19) | .18 |
| Duration of chronic disease |  | |  |  |  |
| ≤5 |  | |  |  |  |
| >5 to ≤10 | 0.36 (0.16-0.84) | | .02 | 1.09 (0.56-2.13) | .80 |
| >10 to ≤18 | 0.52 (0.21-1.29) | | .16 | 2.55 (1.21-5.37) | .01 |
| >18 | 0.61 (0.25-1.49) | | .28 | 1.45 (0.70-3.01) | .31 |
| At least one admission to hospital due to chronic disease |  | |  |  |  |
| No |  | |  |  |  |
| Yes | 0.30 (0.13-0.67) | | .004 | 0.63 (0.35-1.13) | .12 |
| The use of the Internet without help of other persons |  | |  |  |  |
| No |  | |  |  |  |
| Yes | 0.97 (0.42-2.24) | | .93 | 1.05 (0.48-2.26) | .91 |
| Opinion about the usefulness of the Internet in helping to make decision about own health |  | |  |  |  |
| Not useful at all |  | |  |  |  |
| Not useful | 4.68 (1.06-20.79) | | .04 | 1.92 (0.54-6.79) | .31 |
| Unsure | 3.74 (1.01-13.90) | | .04 | 1.20 (0.39-3.65) | .75 |
| Useful | 12.87 (3.42-48.38) | | <.001 | 3.05 (1.04-8.94) | .04 |
| Very useful | 15.02 (3.37-66.94) | | <.001 | 3.69 (1.11-12.31) | .03 |
